# Geological and Geochemical Controls on Subsurface Microbial Life in the Samail Ophiolite, Oman

**DOI:** 10.3389/fmicb.2017.00056

**Published:** 2017-02-07

**Authors:** Kaitlin R. Rempfert, Hannah M. Miller, Nicolas Bompard, Daniel Nothaft, Juerg M. Matter, Peter Kelemen, Noah Fierer, Alexis S. Templeton

**Affiliations:** ^1^Department of Geological Sciences, University of ColoradoBoulder, CO, USA; ^2^National Oceanography Centre, University of SouthamptonSouthampton, UK; ^3^Lamont-Doherty Earth Observatory, Columbia UniversityPalisades, NY, USA; ^4^Cooperate Institute for Research in Environmental Sciences, University of ColoradoBoulder, CO, USA; ^5^Department of Ecology and Evolutionary Biology, University of ColoradoBoulder, CO, USA

**Keywords:** serpentinization, water-rock interactions, deep subsurface biosphere, Samail Ophiolite, gabbro, peridotite, hyperalkaline

## Abstract

Microbial abundance and diversity in deep subsurface environments is dependent upon the availability of energy and carbon. However, supplies of oxidants and reductants capable of sustaining life within mafic and ultramafic continental aquifers undergoing low-temperature water-rock reaction are relatively unknown. We conducted an extensive analysis of the geochemistry and microbial communities recovered from fluids sampled from boreholes hosted in peridotite and gabbro in the Tayin block of the Samail Ophiolite in the Sultanate of Oman. The geochemical compositions of subsurface fluids in the ophiolite are highly variable, reflecting differences in host rock composition and the extent of fluid-rock interaction. Principal component analysis of fluid geochemistry and geologic context indicate the presence of at least four fluid types in the Samail Ophiolite (“gabbro,” “alkaline peridotite,” “hyperalkaline peridotite,” and “gabbro/peridotite contact”) that vary strongly in pH and the concentrations of H_2_, CH_4_, Ca^2+^, Mg^2+^, ${\text{NO}}_{3}^{-}$, ${\text{SO}}_{4}^{2-}$, trace metals, and DIC. Geochemistry of fluids is strongly correlated with microbial community composition; similar microbial assemblages group according to fluid type. Hyperalkaline fluids exhibit low diversity and are dominated by taxa related to the Deinococcus-Thermus genus *Meiothermus*, candidate phyla OP1, and the family Thermodesulfovibrionaceae. Gabbro- and alkaline peridotite- aquifers harbor more diverse communities and contain abundant microbial taxa affiliated with *Nitrospira*, Nitrosospharaceae, OP3, Parvarcheota, and OP1 order Acetothermales. Wells that sit at the contact between gabbro and peridotite host microbial communities distinct from all other fluid types, with an enrichment in betaproteobacterial taxa. Together the taxonomic information and geochemical data suggest that several metabolisms may be operative in subsurface fluids, including methanogenesis, acetogenesis, and fermentation, as well as the oxidation of methane, hydrogen and small molecular weight organic acids utilizing nitrate and sulfate as electron acceptors. Dynamic nitrogen cycling may be especially prevalent in gabbro and alkaline peridotite fluids. These data suggest water-rock reaction, as controlled by lithology and hydrogeology, constrains the distribution of life in terrestrial ophiolites.

## Introduction

Terrestrial deep subsurface environments contain a significant microbial biosphere (Whitman et al., 1998). Recent estimates suggest microbial biomass in the continental subsurface constitutes up to 10^16^–10^17^ g C, or 2–19% of Earth's total biomass (McMahon and Parnell, 2014). Although expansive, the rock-hosted terrestrial deep biosphere is assumed to be inherently energy limited (Teske et al., 2013; Parnell and McMahon, 2016). Subsurface ecosystems often experience states of low energy fluxes such that biomass turnover times are estimated in thousands of years (Lomstein et al., 2012; Hoehler and Jørgensen, 2013). Due to the isolation of deep subsurface environments from the atmosphere and meteoric water cycle, these environments are commonly anoxic and oligotrophic. Even in the shallow subsurface, oxygen is typically depleted, restricting energy metabolism to primarily anaerobic respiration and fermentation (Lovley and Chapelle, 1995). Microorganisms that survive in these isolated waters are adapted to utilize endogenous energy sources or rely on exogenous fluxes of energy, even if these fluxes are slow or sporadic (Kieft et al., 2005).

Water-rock reactions are one possible endogenous, long-lasting source of energy capable of supporting chemoautotrophic life in the terrestrial subsurface. Molecular hydrogen (H_2_) can be produced in deep terrestrial systems via abiotic water-mineral reactions such as radiolysis of water or the hydration and oxidation of iron silicates (Lin et al., 2005a,b; McCollom and Bach, 2009; Sherwood Lollar et al., 2014). Abundant H_2_ has been detected in many deep continental bedrock systems, including the aquifers of the Fennoscandian Shield, the Witwatersrand basin in South Africa, and the Canadian Shield (Pedersen, 1997; Kieft et al., 2005; Lin et al., 2006; Sherwood Lollar et al., 2007, 2014). These systems host microbial communities inferred to utilize diverse metabolisms including H_2_ or methane oxidation, as well as the reduction of nitrate, sulfate, iron, or carbon dioxide (Pedersen, 1997, 2012; Moser et al., 2003; Sherwood Lollar et al., 2007; Lau et al., 2014; Kieft, 2016; Wu et al., 2016).

The availability of electron donors and acceptors for subsurface microorganisms may be strongly influenced by host-rock composition; however, few hard-rock geologic settings that can support microbial ecosystems have been explored. Ultramafic rocks in tectonically exposed massifs of mantle peridotite, particularly in terrestrial ophiolites and seafloor outcrops surrounding slow-spreading mid-ocean ridges, have great potential to harbor an active subsurface biosphere through serpentinization reactions that could yield not only hydrogen, but also organic carbon (Schulte et al., 2006; Schrenk et al., 2013). Aqueous alteration of olivine and pyroxene produces serpentine phases (serpentinization) and other secondary minerals as well as highly reduced, hyperalkaline fluids (Barnes and O'Neil, 1969; Bruni et al., 2002; McCollom and Bach, 2009; Paukert et al., 2012). Under these conditions, the oxidation of iron coupled to the reduction of water commonly generates hydrogen (Frost, 1985; Mayhew et al., 2013) as well as low-weight organic acids such as formate and acetate through the reduction of dissolved inorganic carbon (McCollom and Seewald, 2003, 2007). The formation of hydrocarbons such as methane is relatively more kinetically inhibited, but may occur in fluids of longer residence time, or in gas phase reactions on the surface of mineral catalysts (Sherwood Lollar et al., 1993; Etiope et al., 2011, 2013a,b; Etiope and Ionescu, 2015; McCollom, 2016). The hydrogen and reduced carbon compounds produced from these reactions can act as electron donors to power microbial ecosystems (Karl, 1995; Sleep et al., 2004; Proskurowski et al., 2008; Russell et al., 2010; Etiope et al., 2015; McCollom, 2013; Miller et al., 2016).

Several recent investigations have identified microbial communities inhabiting terrestrial serpentinite systems at surface springs (Brazelton et al., 2013; Suzuki et al., 2013; Tiago and Veríssimo, 2013; Morrill et al., 2014; Cardace et al., 2015; Meyer-Dombard et al., 2015; Woycheese et al., 2015), but little is known regarding the organisms that persistently inhabit the subsurface of these systems. The microbial community composition of surface seeps could be significantly altered from that of the subsurface by the introduction of atmospheric oxidants and carbon. By understanding the mechanisms by which subsurface microbial communities survive in isolation from the atmosphere, we can develop a better understanding of the limits to life in the continental subsurface and on Earth.

The direct exploration of the habitability of subsurface terrestrial serpentinizing systems has been hindered in part due to sampling difficulties. The Samail Ophiolite in the Sultanate of Oman provides a unique setting to investigate subsurface serpentinite-hosted ecosystems because a series of pre-existing boreholes, drilled and monitored by the Ministry of Regional Municipalities and Water Resources, allows for access to subsurface fluids. The Samail Ophiolite is the largest and best exposed of all ophiolites, providing a complete cross-section through about 7 km of oceanic crust and 15 km of underlying, upper mantle rocks for study (Boudier and Coleman, 1981; Coleman and Hopson, 1981; Lippard et al., 1986; Glennie, 1996; Nicolas et al., 2000). Here, over 15,000 km^3^ of partially serpentinized peridotite in the ophiolite is actively undergoing hydration and carbonation reactions at temperatures estimated at ≤60°C, producing hyperalkaline fluids enriched in dissolved hydrogen and methane in the subsurface (Barnes et al., 1978; Neal and Stanger, 1983, 1985; Clark and Fontes, 1990; Kelemen and Matter, 2008; Kelemen et al., 2011, 2013; Paukert et al., 2012; Streit et al., 2012; Miller et al., 2016). The unique size, exposure, and accessibility of the Samail Ophiolite allows for sampling across a broad range of geologic and hydrologic settings to fully inventory the diversity of the subsurface biosphere.

This study explores microbial diversity within the subsurface of an ophiolite that is actively undergoing low-temperature water/rock reaction. Through preexisting boreholes in the Samail Ophiolite, we had access to deep fluid samples from gabbro and peridotite-hosted aquifers. This allowed us to directly investigate fluid geochemistry and the phylogenetic diversity of subsurface microbial communities *in situ*, as well as infer putative metabolisms that could be operative in this environment. We integrate geochemical and microbial data to provide first insights into how microbial community composition varies within terrestrial ophiolites as controlled by local geology.

## Methods

### Site description

**Figure 1:**
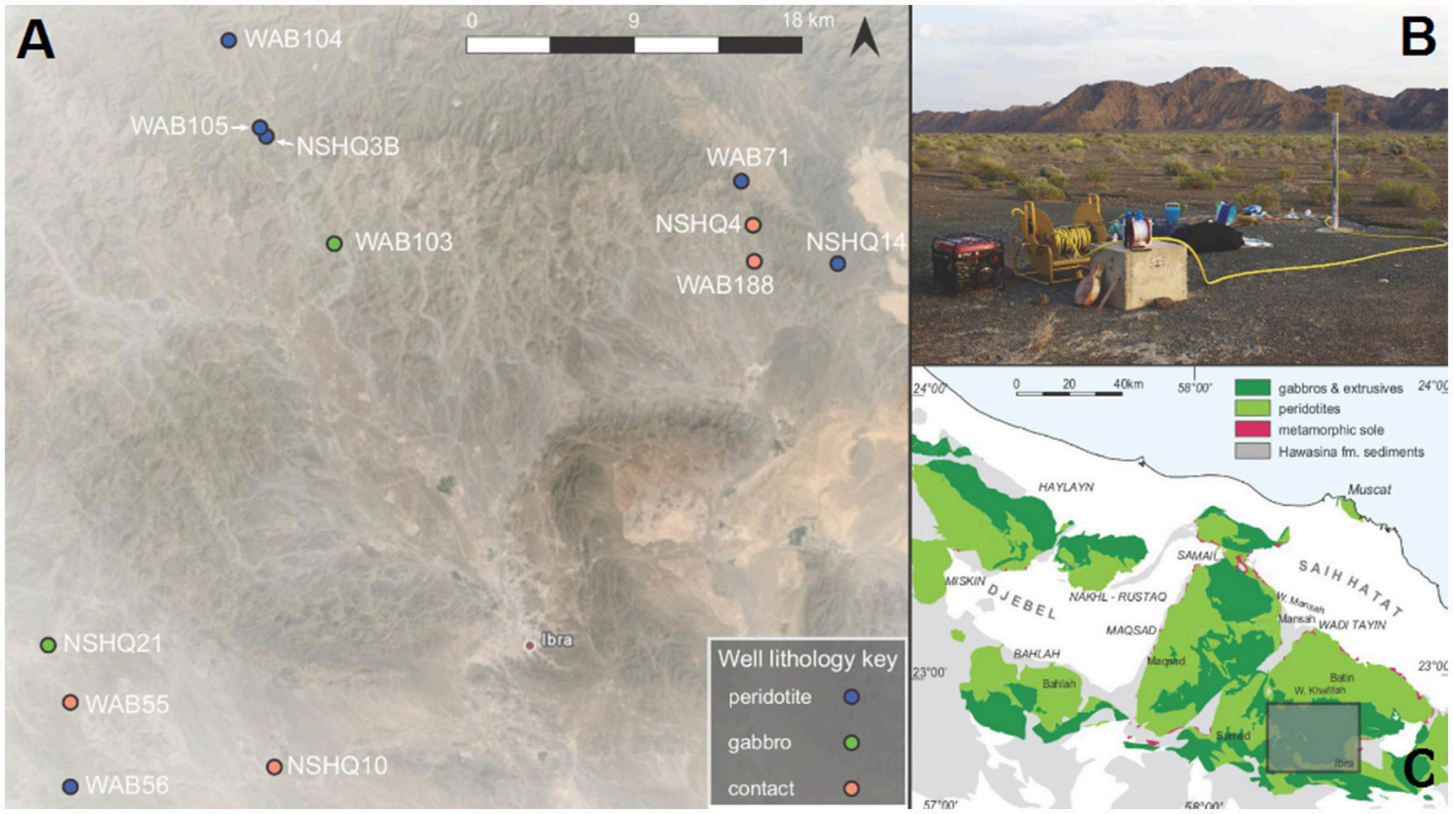
**A submersible pump (B)** was used to retrieve 20 deep fluid samples from a subset of 12 Omani ministry wells **(A)** that span the crust-mantle boundary in the northern Sharquyah region of the Samail Ophiolite **(C)**. For geographical coordinates for wells, see Table 1. Map data for **(C)** are from Nicolas et al. (2000); map is modified from the Oman Drilling Project http://www.omandrilling.ac.uk/.

In January 2014, January 2015, and February 2016, fluids were obtained from deep wells (up to ~475 m deep) previously drilled in peridotite and gabbro for the Oman Ministry of Regional Municipalities and Water Resources. Over the course of these three field seasons, 20 water samples, including particulates and dissolved gases, were collected from a total of 12 wells. Geographic coordinates, elevations, and depths of boreholes are reported in Table 1; all wells were drilled vertically (90 degrees). A map of sampling sites is provided in Figure 1. This series of wells spans the crust/mantle transition of the Samail Ophiolite in the Tayin block (Figure 1). These wells access the water table, and they commonly intersect anoxic fluids that have extensively reacted with mafic to ultramafic rocks. We classified wells as belonging to one of three lithologies (peridotite, gabbro, or contact) based on field observations of surrounding geology (described in Table 1). Wells that were classified as “contact” sit in gabbro or peridotite, but are in close proximity (<1 km) to the surface boundary between crustal and mantle rocks. Large changes in rock permeability may occur at these boundaries, giving rise to the potential injection of hyperalkaline fluids into adjacent higher permeability gabbros (Dewandel et al., 2005). Faulting at the crust-mantle boundary has also been documented (Boudier and Coleman, 1981; Nicolas et al., 2000), which may facilitate the mixing of gabbro- and peridotite-reacted fluids.

**Table 1:** **Well location, depth, elevation, and geology along with measurements of water level and pump depth at the time of sampling**.

**Well**	**UTM easting**	**UTM northing**	**Geologic description**	**Lithology**	**Elevation mabsl**	**Well depth m**	**Year**	**Water level mbtc**	**Pump mbtc**
NSHQ3B	645,068	2,536,069	Borehole in wadi alluvium surrounded by harzburgite	Peridotite	688	472	2015	2.3	20
NSHQ14	675,495	2,529,716	Harzburgite	Peridotite	526	304	2014	7.7	18
							2015	10	20
							2016	8.8	70
WAB56	634,851	2,501,617	Harzburgite	Peridotite	519	106	2015	6.7	12
							2016	7.6	50
WAB71	670,322	2,533,981	Harzburgite-Dunite	Peridotite	608	136.5	2015	7.8	18
							2016	8.2	50
WAB104	643,099	2,541,124	Harzburgite	Peridotite	842	120.4	2016	32.9	70
WAB105	644,678	2,536,524	Harzburgite	Peridotite	738	120.5	2016	15.24	50
NSHQ21	633,569	2,509,105	Gabbro	Gabbro	514	233	2015	3.33	20
WAB103	648,577	2,530,362	Gabbro	Gabbro	632	101	2015	15	22
							2016	15.24	50
NSHQ4	670,971	2,531,699	Harzburgite; near fault	Contact	543	304	2014	2.6	18
							2015	5	22
NSHQ10	645,706	2,502,793	Cumulate peridotite, near contact with gabbro	Contact	453	403	2016	14.3	50
WAB55	634,777	2,506,101	Harzburgite with carbonated veins, near contact with gabbro	Contact	531	102	2015	5.76	18
							2016	6.7	50
WAB188	671,123	2,529,798	Gabbro, near contact with harzburgite	Contact	514	78	2015	9.2	20
							2016	7.9	30

*Geographic coordinates are provided in the UTM coordinate system for zone 40Q*.

### Field methods

A submersible pump was utilized to collect subsurface fluids from boreholes for geochemical and microbiological analyses. The depth of the water table and depth of pumping for sample retrieval are reported in Table 1. Each well was initially pumped for at least 20 minutes with the goal of flushing 1-3 volumes of well water prior to sampling. Fluid temperature, conductivity, dissolved oxygen, pH, and oxidation-reduction potential (Eh) were measured in the field using a WTW 3400i multi-parameter field meter calibrated using Fisher Scientific pH 7 and 10 buffers (analytical error of ± 0.1 units for pH, ± 1 mV for Eh, ± 0.1°C for temperature, and ±0.003 mM dissolved oxygen). Sampling commenced once fluid pH and conductivity measurements stabilized after flushing. Conductivity measurements were utilized solely as an in-field measure of chemical stability. The reactivity of fluids induces mineral precipitation upon contact with the atmosphere, leading to a large uncertainty in probe measurements. Accordingly, conservative geochemical measurements, such as chloride concentration, are more accurate for comparing fluids.

Redox sensitive species (ferrous iron, nitrite) were measured in the field using Hach water chemistry test ampules (Hach Company, Loveland, CO) with a portable Hach DR/890 colorimeter (analytical error for ferrous iron ± 0.00016 mM, nitrite ± 0.000065 mM). Numerous subsamples of deep fluids were also prepared in-field for laboratory-based geochemical analysis. Aliquots of fluid were collected in sterile syringes, filtered through a 0.22 micron filter, and injected into nitrogen-purged or evacuated vials with butyl stoppers for headspace gas analysis. Fluids for organic acid measurements were filtered through a 0.22 micron filter to remove microbial cells. The filters were pre-purged with fluid before sample collection and the filtrate was collected in ashed, organic-free vials capped with silicon septa. All vials were acid washed, autoclaved, and muffled at 400°C prior to field work. Samples for ICP analysis of major cations and trace elements were filtered with 0.2 micron Millipore filters and acidified in the field with concentrated nitric acid to a pH <2, whereas aliquots for IC analysis of anions were filtered, but preserved unacidified. Samples for dissolved inorganic carbon measurements were poisoned with HgCl_2_ to prevent microbial modification of samples post-collection. Fluids (5–20 L) for biomass recovery were pumped and filtered through 0.2 micron Millipore polycarbonate inline filters to concentrate cells for DNA extraction. Filters were placed in sterile cryovials, transported frozen in liquid nitrogen, and stored in a −80°C freezer until extraction.

### Laboratory methods

Major cations (Na^+^, Mg^2+^, Ca^2+^, Al^3+^) were analyzed using Inductively Coupled Plasma-Mass Spectrometry (ICP-MS) on a Thermoscientific X Series 2 spectrometer (analytical error of ~1%). Trace element (Ni, As, Se, Cu, Cd, Zn, Co, Cr) measurements for 2014 and 2015 fluid samples were conducted using ICP-MS on a Perkin-Elmer Elan DRC-E mass spectrometer according to the U.S. Environmental Protection Agency (EPA) method 6020 (analytical error of ~1%). In 2016, only Ni, Cu, and Cr were measured using ICP-MS as described above for major cation analysis on the X Series 2 spectrometer (analytical error ~2%). Silica concentrations were determined using Inductively Coupled Plasma-Optical Emission Spectrometry (ICP-OES) on a 3410 Applied Research Laboratories instrument following EPA method 6010 (analytical error of ~2%); for 2016 samples, the American Standard Test Method D 859-00 for colorometric measurement of silica was utilized with a Beckman Coulter DU 730 UV/Vis spectrophotometer (analytical error of 1%). Major anions (F^−^, Cl^−^, Br^−^, ${\text{SO}}_{4}^{2-}$, ${\text{NO}}_{3}^{-}$) were measured on filtered samples using ion chromatography (IC) on a Dionex IC25 chromatograph with an AS9-HC IonPac column, with the exception of nitrate, which was measured on a Dionex 4500I chromatograph with an IonPac AS14 column using EPA method 300.0 (analytical error ~2%).

Dissolved inorganic carbon analyses were conducted by SGS United Kingdom Ltd. using the BS EN1484 method on a Shimadzu TOC analyzer (analytical error of 1.5%). Samples for organic acid measurements were sent to NASA Ames Research Center for measurement on a Shimadzu Prominence LC20AT high-performance liquid chromatograph (HPLC) equipped with a SPD-M20A photodiodearray detector (analytical error of 5%).

The concentrations of hydrogen and methane gas exsolved from well fluids were determined using a SRI 8610C gas chromatograph (GC) with a 2 m by 1 mm ID micropacked ShinCarbon ST column with nitrogen as the carrier gas. 0.5 mL of headspace gas from butyl stopper-capped fluid samples were injected into the sampling port on the GC for analysis. Hydrogen and methane were measured concurrently using a thermal conductivity detector (TCD) and a flame ionization detector (FID) respectively (detection limit of 10 ppm and analytical error of 5%).

### 16S rRNA sequencing and analysis

DNA was extracted from a one quarter subsample of each filter using the MoBio PowerSoil kit (MoBio Inc., Carlsbad, CA) according to the manufacturer's instructions. PCR amplification of the V4-V5 region of the 16S rRNA gene was performed in triplicate using barcoded 515F and 806R primers that contained the appropriate Illumina adapters and linkers according to the protocol described by Caporaso et al. (2012). Triplicate reactions were performed using 1 μL of extracted DNA and resulting amplicons were composited for each sample. The concentration of amplicons was determined using the PicoGreen dsDNA assay. Equimolar concentrations of amplicons from all samples were pooled together for sequencing. Sequencing was conducted on an Illumina MiSeq at the University of Colorado Next-Generation Sequencing Facility following the 2 × 250 bp paired-end protocol. Reads were processed according to the protocol described by Barberán et al. (2015). In short, sequences were demultiplexed using a custom Python script (https://github.com/leffj/helper-code-for-uparse). Merging, quality filtering, and phylotype clustering were conducted using the UPARSE pipeline (Edgar, 2013). Reads were quality filtered using a maximum *e*-value of 0.5, sequences were dereplicated, and singletons were removed. Reads were clustered into phylotypes using default settings to create a *de novo* database. Raw sequences were then mapped globally to the *de novo* database at the 97% similarity threshold. Taxonomy was assigned using the RDP classifier trained on the Greengenes 13_8 database with a confidence threshold of 0.5 (Wang et al., 2007; McDonald et al., 2012). Samples were rarefied to 15,000 sequences prior to downstream analyses. All sequences are accessible on the Short Read Archive (NCBI) database under accession SRP092764.

### Statistical analyses

All multivariate statistical analyses were performed in R (R Development Core Team, 2015). Principal component analysis (PCA) of environmental data was performed through singular value decomposition of the centered correlation matrix using the “prcomp” function in the “stats” package and plotted with the package “ggbiplot.” Calculated environmental loadings and site scores are listed in Supplemental Table 1. For microbial community comparisons, rarefied OTU tables were Hellinger-transformed and Bray-Curtis dissimilarity matrices were generated. We visualized these results with a non-metric multidimensional scaling (NMDS) plot using ANOSIM to test if there was a significant difference in the microbial communities based on classified fluid chemistries. The ordination and ANOSIM statistic was implemented with the package “vegan.” Correlation of the microbial assemblages with geochemistry was tested using a Mantel test of the Hellinger-transformed Bray-Curtis dissimilarity matrix of microbial community composition and a standardized distance matrix of measured environmental parameters using the functions “decostand” and “mantel” in the package “vegan.”

## Results

### Aqueous geochemistry of subsurface fluids

**Figure 2:**
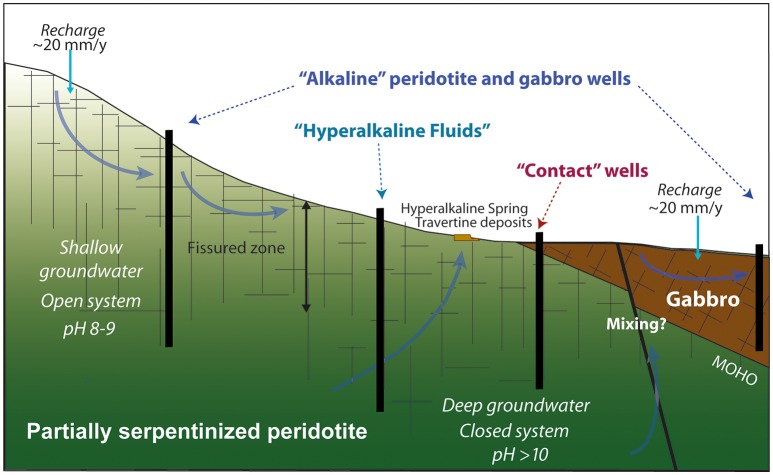
**Conceptual model of the origin of *hyperalkaline peridotite*, *alkaline peridotite*, *gabbro*, and *contact* fluids in the aquifers of the Samail Ophiolite**. (Figure adapted from Kelemen et al., 2014).

The aqueous chemistry of sampled fluids reflects the diversity of geologic and hydrologic settings sampled. Fluids were collected from a series of deep wells drilled into crustal gabbro, mantle peridotite, or the crust-mantle transition zone. These wells access fluids of varying depth and flow path, which likely correspond to differences in residence time and the extent of fluid-rock interaction, as depicted in Figure 2.

Field measurements (see Table 2) demonstrate highly variable subsurface fluid compositions as evidenced by pH (7.4–11.4) and redox state (Eh of −546 to +180 mV). Potential substrates for microbial metabolism differ in concentration considerably between wells. Sulfate is always present, ranging from 0.01 to 3.9 mM. Nitrate and nitrite are sometimes below detection, but can reach values as high as 0.36 and 0.010 mM respectively. Aqueous ferrous iron, methane and hydrogen concentrations range from below detection limit to 0.029, 2.3, and 2.9 mM. Measured dissolved inorganic carbon (DIC) varies considerably well to well (0.18–2.6 mM). We also tested for the presence of low molecular weight organic acids during the 2015 field season, which were detected in μM concentrations in all nine wells sampled. Acetate (0.47–4.4 μM) and formate (1.0–2.3 μM) were detected in all wells; lactate, propionate, butyrate, and valerate were only measured above detection limit in a few of the sampled wells (Table 2).

**Table 2:** **Measurements of major and minor elements, organics, and dissolved gases, along with pH, temperature, conductivity, and redox potential of well fluids**.

	**NSHQ3B**	**NSHQ14**			**WAB56**		**WAB71**		**WAB104**	**WAB105**	**NSHQ21**	**WAB103**		**NSHQ4**		**NSHQ10**	**WAB55**		**WAB188**		**Detection limit**	
	**Peridotite**	**Peridotite**			**Peridotite**		**Peridotite**		**Peridotite**	**Peridotite**	**Gabbro**	**Gabbro**		**Contact**		**Contact**	**Contact**		**Contact**		**In**	
	**2015**	**2014**	**2015**	**2016**	**2015**	**2016**	**2015**	**2016**	**2016**	**2016**	**2015**	**2015**	**2016**	**2014**	**2015**	**2016**	**2015**	**2016**	**2015**	**2016**	μ**M**	
pH	8.4	11.4	11.3	11.2	10.6	11.0	11.0	11.1	8.5	8.3	7.4	8.2	8.2	10.6	10.5	8.8	9.3	9.2	8.7	7.6	–	
Temperature °C	30.1	34.9	35.3	35.8	35.1	–	33.1	34.5	33.4	31.6	33.7	30.4	33.8	33.3	33.3	–	30	34.7	34.2	33	–	
Conductivity (uS/cm)	297	3199	1716	2525	354	930	1109	1803	493	448	1905	1095	1448	2386	1249	–	585	1171	427	926	–	
Eh (mV)	158	−32	−307	−252	−251	−546	−126	−86	180	178	172	130	167	−103	−342	–	78	110	−220	139	–	
Na^+^ (mM)	0.626	12.1	11.2	9.39	3.07	3.56	5.61	5.20	0.661	0.591	17.7	11.9	10.9	6.75	8.09	4.56	3.48	4.15	2.51	2.55	1.7	
Ca^2+^ (mM)	0.303	3.69	3.42	3.78	0.430	0.543	3.82	3.78	0.115	0.254	1.88	0.419	0.364	5.16	4.13	0.175	0.074	0.083	0.717	1.10	0.15	
Mg^2+^ (mM)	1.66	0.005	0.001	0.002	dL	0.001	0.001	0.001	1.89	1.58	2.46	0.236	0.238	0.620	0.003	0.373	3.14	2.74	1.52	1.64	0.21	
SiO_2_(aq) (mM)	0.247	0.007	0.005	0.005	0.198	0.172	0.017	0.024	0.210	0.043	0.782	0.480	0.848	0.010	0.015	0.969	0.006	0.006	0.232	0.785	0.67	0.33^a^
Cl^−^ (mM)	0.88	16.6	16.6	15.8	3.2	3.5	12.5	12.9	0.75	0.77	6.2	7.6	7.4	14.6	18.2	0.78	14.8	7.3	3.6	3.5	13.3	
Br^−^ (mM)	0.001	0.028	0.025	0.022	0.001	0.003	0.014	0.015	0.001	0.001	0.004	0.012	0.010	0.010	0.019	0.001	0.012	0.007	0.003	0.003	0.18	
Al^3+^ (mM)	dL	0.0002	0.0001	dL	0.0006	0.0010	0.0012	0.0007	dL	dL	0.0001	0.0005	0.0001	dL	0.0009	0.0003	0.0001	0.0003	0.0001	dL	0.07	
Fe^2+^ (mM)	dL	–	0.009	0.029	dL	0.009	0.013	0.035	dL	dL	dL	dL	0.002	–	0.024	dL	dL	–	0.002	dL	0.36	
																						
${\text{SO}}_{4}^{2-}$ (mM)	0.28	0.08	0.15	0.09	0.02	0.01	0.03	0.05	0.50	0.29	0.78	1.7	1.8	0.22	0.36	0.19	3.9	0.90	0.59	1.2	1.2	
${\text{NO}}_{3}^{-}$ (mM)	0.11	0.02	0.02	0.00	0.03	0.00	dL	dL	0.15	0.14	0.26	0.36	0.34	0.01	0.02	0.08	0.15	0.15	0.10	0.15	8.1	
${\text{NO}}_{2}^{-}$ (mM)	0.004	–	0.0004	dL	0.001	0.003	0.001	0.010	0.011	0.004	0.001	0.001	0.003	–	dL	0.004	0.003	–	dL	0.003	0.03	
dO (mM)	0.17	–	0.01	–	0.02	–	0.01	–	–	–	0.03	0.04	–	–	0.00	–	0.09	–	0.004	–	0.63	
																						
DIC (mM)	2.5	–	0.19	–	0.22	–	0.21	–	–	–	2.6	2.3	–	–	0.18	–	2.6	–	2.3	–	0.10	
H_2_ (mM)	dL	0.67	2.9	0.22	dL	dL	dL	dL	dL	dL	dL	dL	dL	0.18	dL	dL	dL	–	dL	dL	0.45^*^	
CH_4_ (mM)	dL	0.17	0.12	0.05	0.01	dL	0.02	0.02	dL	dL	dL	dL	dL	1.4	2.3	dL	dL	–	0.04	dL	0.45^*^	
																						
lactate (μM)	dL	–	0.28	–	dL	–	dL	–	–	–	dL	dL	–	–	dL	–	dL	–	dL	–	0.24	
acetate (μM)	0.47	–	1.2	–	4.4	–	0.63	–	–	–	0.49	0.43	–	–	1.4	–	2.0	–	3.8	–	0.07	
formate (μM)	1.2	–	1.7	–	1.5	–	1.5	–	–	–	1.1	1.1	–	–	2.3	–	1.4	–	1.0	–	0.24	
propionate (μM)	dL	–	0.26	–	0.30	–	0.23	–	–	–	dL	dL	–	–	0.05	–	0.15	–	0.04	–	0.01	
butyrate (μM)	dL	–	0.23	–	dL	–	dL	–	–	–	dL	dL	–	–	0.30	–	dL	–	0.20	–	0.16	
valerate (μM)	dL	–	dL	–	dL	–	dL	–	–	–	dL	dL	–	–	0.05	–	dL	–	dL	–	0.03	
Ni (μM)	0.036	0.137	0.199	dL	0.045	dL	0.216	dL	0.006	0.008	0.161	0.038	0.005	0.150	0.264	0.027	0.041	0.005	0.060	0.050	0.0002	0.0017^b^
As (μM)	0.005	0.040	0.050	–	0.007	–	0.036	–	–	–	0.061	0.022	–	0.040	0.058	–	0.016	–	0.016	–	0.0004	
Se (μM)	0.017	0.144	0.171	–	0.014	–	0.087	–	–	–	0.198	0.230	–	0.090	0.127	–	0.064	–	0.040	–	0.002	
Cu (μM)	0.011	0.040	0.098	0.001	0.028	0.005	0.048	0.001	0.013	0.007	0.151	0.106	0.011	0.030	0.080	0.002	0.046	0.010	0.042	0.002	0.0002	0.00002^b^
Cd (μM)	0.002	dL	dL	–	0.001	–	0.0001	–	–	–	0.0004	0.0002	–	dL	0.008	–	0.045	–	0.003	–	0.0001	
Zn (μM)	0.080	0.056	0.057	–	0.272	–	0.057	–	–	–	0.104	0.102	–	0.030	0.390	–	–	–	0.143	–	0.002	
Co (μM)	0.001	0.005	0.007	–	0.002	–	0.009	–	–	–	0.005	0.001	–	0.010	0.011	–	0.003	–	0.003	–	0.0001	
Cr (μM)	0.112	0.170	0.132	0.011	0.075	0.001	0.170	0.004	0.027	0.031	0.168	0.158	0.019	0.160	0.114	0.017	0.205	0.038	0.084	0.050	0.0009	0.0004^b^

*2014 data was previously reported in Miller et al., 2016 Gas concentrations were determined by measuring the headspace of anaerobic vials filled with site water. DIC concentrations are reported in mM total carbon*.

a *Denotes the detection limit for 2016 silica data measured by spectrophotometry*.

b *Is the detection limit for 2016 trace element data measured on the X-Series2*.

* *Indicates the detection limit for H_2_ and CH_4_ by GC, calculated at STP*.

**Figure 3:**
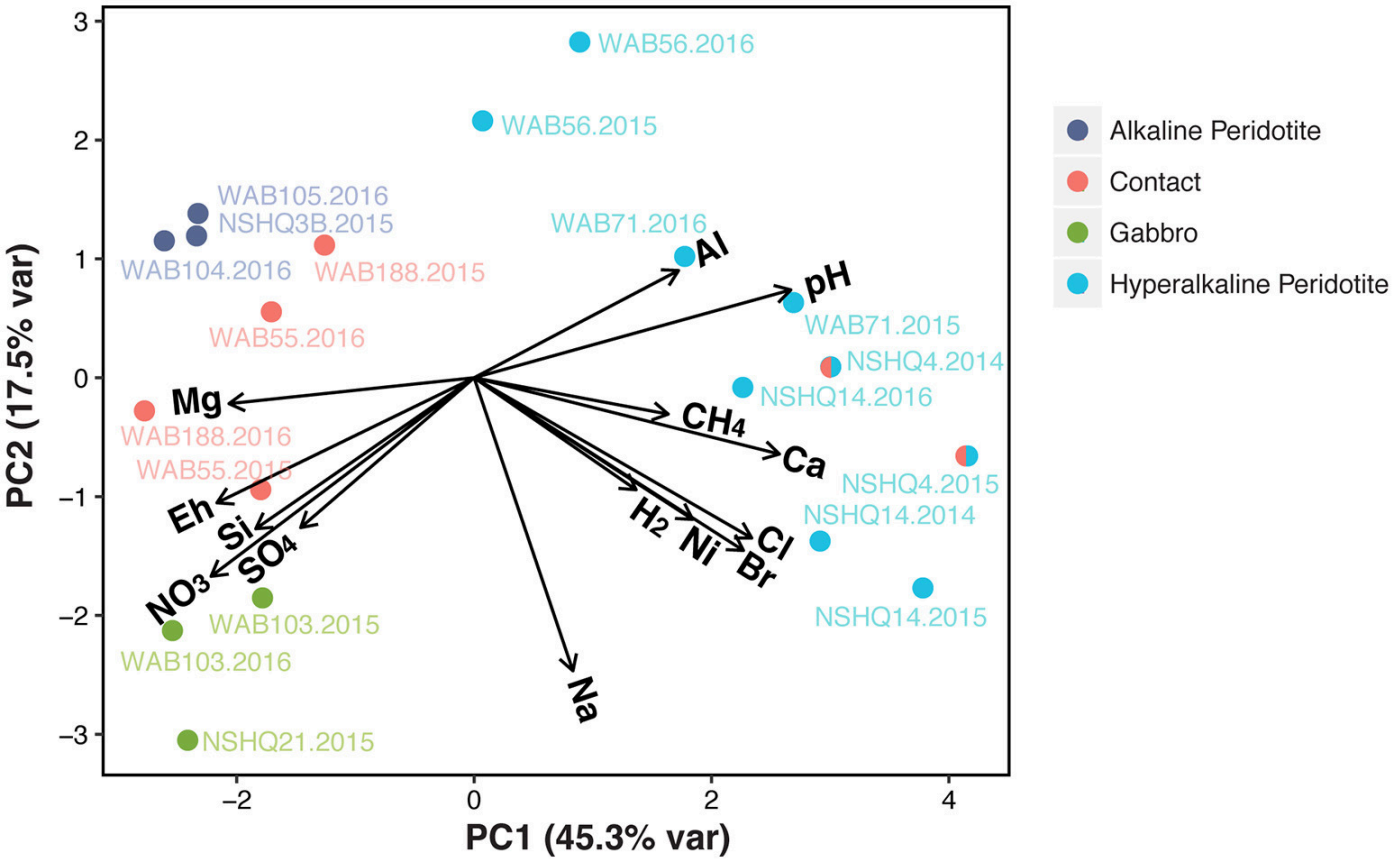
**Principal component analysis of geochemical parameters measured in wells for all three years of sampling**. Calculated variable loadings are plotted as vectors to display the relationship of each variable with the first and second principal components. The well NSHQ4 is depicted as both a contact well and a hyperalkaline peridotite well because this well is close to the crust-mantle boundary, but displays geochemistry consistent with hyperalkaline peridotite fluids.

In order to systematically evaluate the relationship between different wells and subsurface fluid chemistry, we performed a principal component analysis (PCA) of the geochemical parameters measured in all three field seasons (Figure 3). Calculated variable loadings (see Supplemental Table 1) for each chemical constituent were plotted as vectors on the biplot, demonstrating the relationship of the parameter with the first and second components. The first component (PCA 1) explains 45.3% of the variance and appears to be primarily driven by differences in magnesium, calcium, pH, and dissolved methane. The second component (PCA 2) only explains 17.5% of the variance.

The PCA reveals a strong partitioning between the 20 fluid chemistries on the biplot. Notably, we can distinguish between two geochemical subgroups of peridotite-hosted fluids, which we classify as “hyperalkaline” (pH >10) or “alkaline” (pH 8–10) for subsequent microbial community analysis. Hyperalkaline peridotite fluids plot in the right quadrants of the PCA and are characterized by high dissolved hydrogen, methane, and calcium concentrations, and high pH. These fluids are enriched in trace elements such as nickel and depleted in magnesium, sulfate, nitrate, and silica. Redox potential is also very low in these samples. Although not pictured due to the lack of 2016 DIC data, hyperalkaline fluids are all limited in DIC (≤ 0.22 mM; see Supplemental Figure 1). In contrast, fluids from the wells located at the crust/mantle contact, alkaline peridotite wells, and gabbro wells all plot in the left quadrants, with the exception of the contact well NSHQ4. These wells are more oxidized and have relatively high DIC concentrations (≥2.3 mM), no detected hydrogen or methane (with the exception of WAB188 in 2015), and relatively high concentrations of oxidants such as sulfate and nitrate. Gabbro wells exhibit similar chemistry to alkaline peridotite wells, but are characterized by higher silica and sodium concentrations, as well as higher concentrations of oxidants (e.g., sulfate and nitrate). Contact wells display more variability in aqueous geochemistry than wells categorized as gabbro or alkaline peridotite. The well NSHQ4, despite its close proximity to the crust-mantle boundary, plots consistently with hyperalkaline peridotite fluids. Accordingly, we depict this well as hybridized (i.e., both contact and hyperalkaline peridotite).

### Microbial diversity and community composition

**Figure 4:**
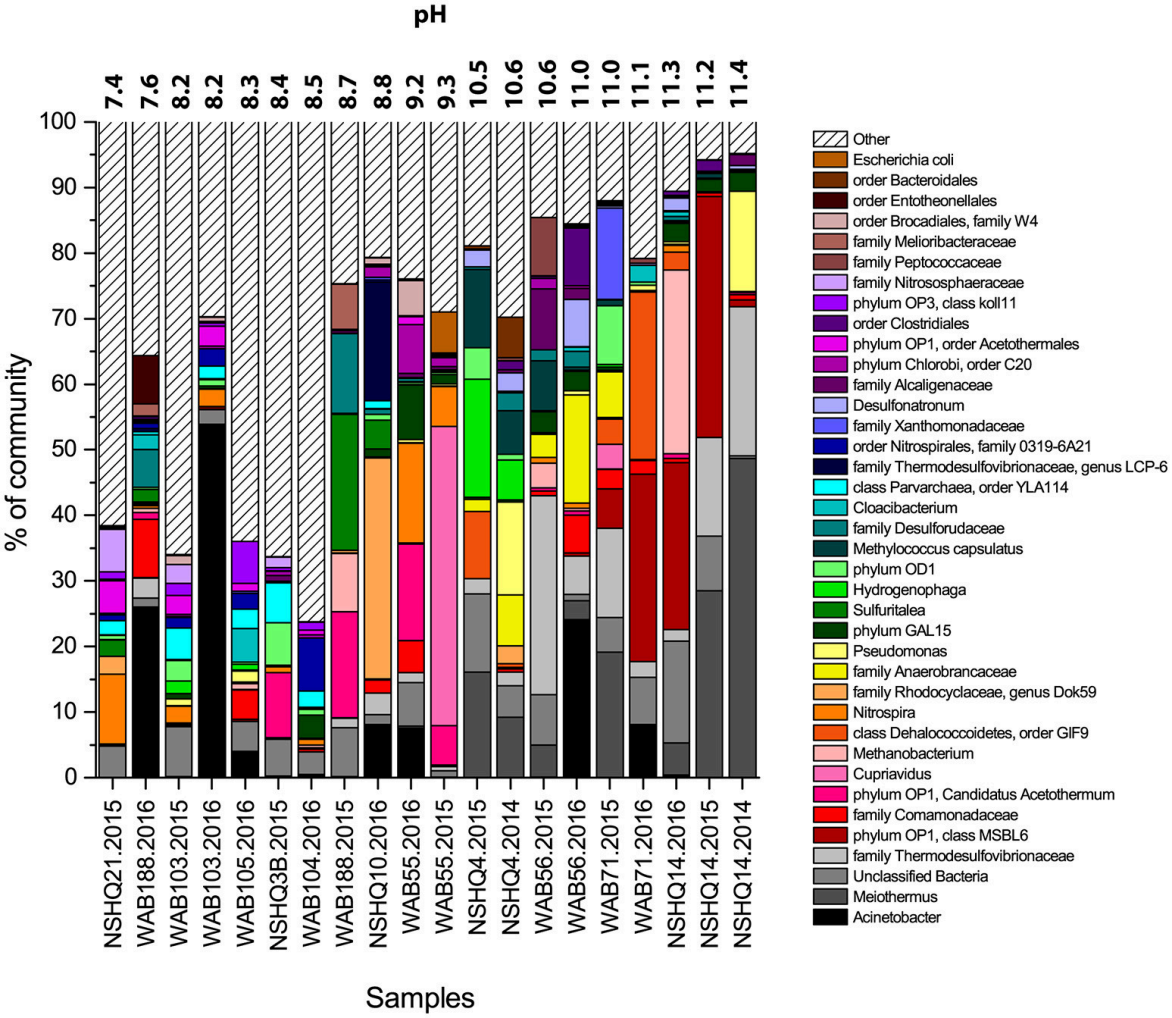
**Microbial community composition of wells, displaying only OTUs that make up at least 5% relative abundance of the detected population in at least one well**. Taxonomy of OTUs is listed to the greatest depth allowed by the confidence threshold set in RDP Classifier. Wells are organized by increasing pH. In high pH wells, most of the diversity can be explained by just a few OTUs.

The microbial community composition for each well was determined through 16S rRNA amplicon sequencing and subsequent taxonomic analysis. A total of 39 abundant (>5% of total reads in a given community) phylotypes were detected in one or more wells (Figure 4, Supplemental Table 2). These 39 phylotypes represent 10 established phyla (8 bacterial, 2 archaeal) and 6 candidate phyla (5 bacterial, 1 archaeal).

Biomass sampled from all wells yielded a greater number of bacterial reads than archaeal reads (Table 3). Archaeal operational taxonomic units (OTUs) comprised just over 15% of the community in only four samples (NSHQ14 in 2016, NSHQ3B, NSHQ21, and WAB103 in 2015). NSHQ14 in 2016 contained sequences that were almost exclusively affiliated with the archaeal genus *Methanobacteria*, constituting 99.1% of the total archaeal reads and 28.0% of total reads for this well. The genus *Methanobacteria* was detected in a total of 16 samples, but was only in high relative abundance (>3%) in two other samples- the crust/mantle contact well WAB188 and the hyperalkaline peridotite well WAB56, both from the 2015 field season. Sequences affiliated with the phyla Thaumarchaeota and Parvarchaea dominated the archaeal populations in all other wells.

**Table 3:** **Overview of 16S amplicon sequencing results**.

**Site**	**Fluid type**	**Year**	**No. of OTUs**	**% Bacteria**	**% Archaea**
NSHQ14	Hyperalkaline Peridotite	2014	84	99.57	0.43
		2015	290	99.93	0.07
		2016	224	71.73	28.27
WAB56	Hyperalkaline Peridotite	2015	151	96.17	3.83
		2016	161	99.63	0.37
WAB71	Hyperalkaline Peridotite	2015	267	95.1	4.9
		2016	153	99.8	0.2
NSHQ3B	Alkaline Peridotite	2015	2364	80.56	19.44
WAB104	Alkaline Peridotite	2016	891	88.64	11.36
WAB105	Alkaline Peridotite	2016	1111	90.75	9.25
NSHQ21	Gabbro	2015	1202	83.33	16.67
WAB103	Gabbro	2015	1776	82.28	17.72
		2016	862	93.44	6.56
NSHQ4	Contact	2014	339	99.25	0.75
		2015	239	99.98	0.02
NSHQ10	Contact	2016	537	95.39	4.61
WAB55	Contact	2015	793	95.41	4.59
		2016	257	98.29	1.71
WAB188	Contact	2015	281	91.11	8.89
		2016	859	95.97	4.03

*The number of operational taxonomic units (OTUs) is reported post rarefaction to 15,000 sequences. Relative percentages of bacteria and archaea were calculated from the assigned taxonomy of OTUs*.

The most abundant bacterial taxa were *Meiothermus* (6.78% of total reads), *Acinetobacter* (6.62%), Thermodesulfovibrionaceae (5.33%), and Candidate Phylum OP1 class MSBL6 (4.94%). Also common were phylum OP1 *Candidatus* Aceothermum (2.53%), *Cupriavidus* (2.48%), Dehalococcoidetes (2.15%), *Nitrospira* (2.14%), and Rhodocyclaceae (genus Dok59, 2.00%). With the exception of *Acinetobacter*, most of these taxa were only abundant in a few individual wells. Microbial community composition was highly variable across wells. Only phylotypes affiliated with candidate phyla OD1, GAL15, and OP1, as well Betaproteobacteria belonging to the family Comamonadaceae were abundant across all samples.

**Figure 5:**
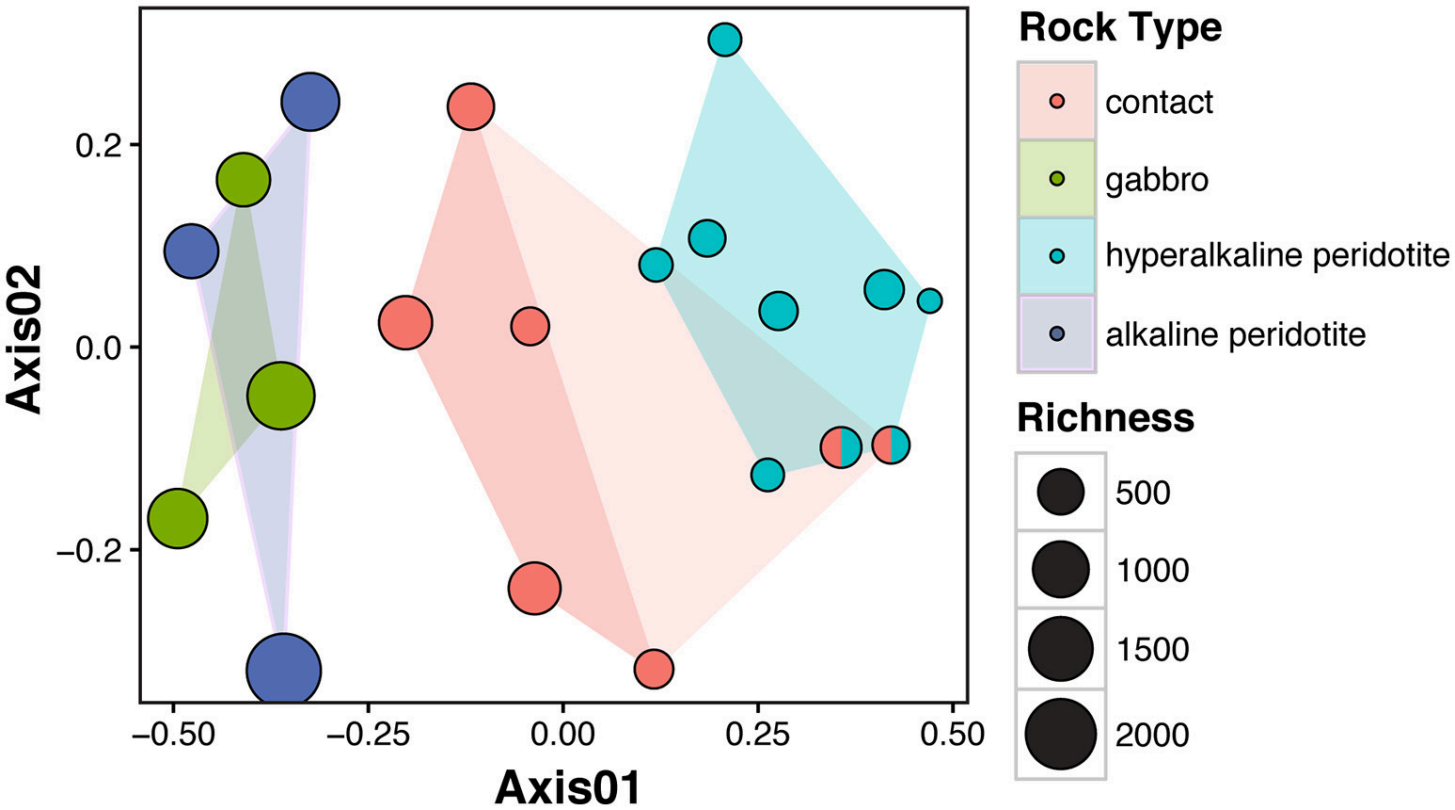
**Non-metric multidimensional scaling (NMDS) analysis of microbial community similarity according to the Bray-Curtis dissimilarity index of rarefied and Hellinger-transformed OTU abundances**. Stress for two-dimensional ordination is 0.14. The well NSHQ4 is depicted as both a contact well and a hyperalkaline peridotite well because this well is located near the crust-mantle boundary, but displays geochemistry consistent with hyperalkaline peridotite fluids. Clustering of microbial communities according to fluid type is statistically supported by ANOSIM (R statistic of 0.85, *P* < 0.001 for fluid type with NSHQ4 as a hyperalkaline peridotite well or R statistic of 0.55, *P* < 0.001 for fluid type with NSHQ4 classified as a contact well).

**Figure 6:**
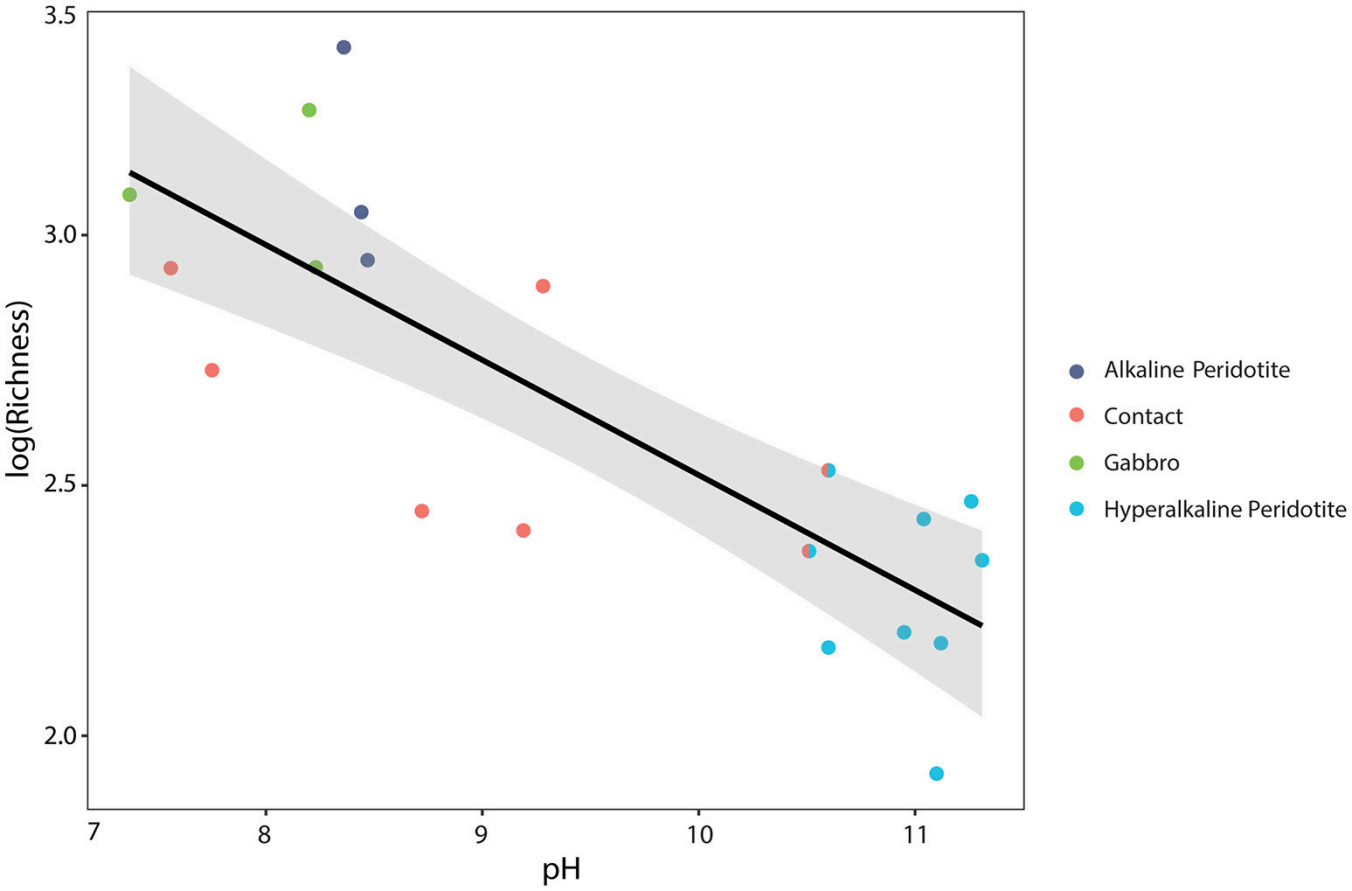
**The base-10 logarithm of richness (as defined as the number of operational taxonomic units in a sample) is negatively correlated with pH**. The calculated linear regression line, log_10_ (Richness) = 4.82–0.23(pH), is plotted in black, with a 95% confidence interval plotted in gray (multiple *r*^2^ = 0.6604, F-statistic = 35.01, *p* < 0.001). Wells are color-coded by fluid-type, with the well NSHQ4 depicted as both a contact well and a hyperalkaline peridotite well because this well is located near the crust-mantle boundary, but displays geochemistry consistent with hyperalkaline peridotite fluids.

Microbial community composition was correlated with fluid chemistry (Mantel test, *r* = 0.43, *p* < 0.001). However, the multi-collinearity of geochemical parameters precludes meaningful statistical correlation of microbial community composition with any one geochemical parameter (see Figure 3). Microbial communities clustered according to the well's fluid type (ANOSIM R statistic of 0.55, *p* < 0.001 for fluid type; Figure 5). The microbial communities of gabbro and alkaline peridotite fluids group together; these wells exhibit the greatest richness of microorganisms (the total number of different OTUs detected per well). In contrast, hyperalkaline peridotite fluids host low diversity microbial communities that plot on the opposite end of the non-metric multidimensional scaling (NMDS) ordination. Crust/mantle contact fluids form a discrete cluster between these two groupings, with the exception of the well NSHQ4 which plots with hyperalkaline peridotite wells. In general, richness appears to be negatively correlated with pH regardless of geologic context (linear regression, multiple *r*^2^ = 0.6604, F-statistic = 35.01, *p* < 0.001; Figure 6).

Hyperalkaline peridotite wells exhibit microbial communities dominated primarily by taxa affiliated with the family Thermodesulfovibrionaceae, candidate phylum OP1 class MSBL6, and *Meiothermus*. In lesser abundance, *Desulfonatronum*, Anaerobrancaceae, *Pseudomonas*, and the order Clostridiales also compose the core microbial community. In addition, taxa within the Dehalococcoidetes, *Methanobacteria, Hydrogenophaga, Methylococcus capsulatus*, Xanthamonadaceae, and Alcaligenaceae groups were found to be abundant in at least one hyperalkaline well. Contact fluids are especially enriched in Betaproteobacteria compared to all other fluid types. In contrast, gabbro and alkaline peridotite fluids are enriched in taxa affiliated with the genus *Nitrospira*, the family 0319-6A-21 (order Nitrospirales), Thaumarcheota belonging to the family Nitrososphaeraceae, candidate phyla OP3 and Parvarcheota, as well as OP1 order Acetothermales.

## Discussion

### Hydrogeochemistry of deep fluids in the samail ophiolite

The chemistry of subsurface fluids in the Samail Ophiolite reflects both differences in host rock as well as hydrologic context—i.e., the extent of equilibration with the host rock or atmosphere and the potential for mixing with other fluids. Groundwater flow in this region follows the local topography from mantle rocks exposed in sharp-peaked mountainous catchments to crustal gabbros downgradient in rounded hills of more moderate relief (Neal and Stanger, 1985; Dewandel et al., 2005). The upper 50 m or more of the Samail Ophiolite is intensely fractured (Neal and Stanger, 1985; Dewandel et al., 2005). The difference in permeability between the upper (<50 m) and lower aquifer from this differential fracturing results in contrasting residence times (Ayraud et al., 2008; Ben Maamar et al., 2015) that should yield distinct hydrochemical regimes: an upper-aquifer, topographic flow regime of more rapid renewal (within the upper 50 m), and a deeper aquifer regime of longer residence time. These flow regimes are depicted in Figure 2 in our proposed conceptual model for groundwater flow in the Samail Ophiolite.

Alkaline peridotite fluids (NSHQ3B, WAB104, WAB105) are likely sourced from near-surface flow in peridotite-hosted aquifers open to the atmosphere. These wells are characterized by high elevations (>650 m above sea level), and thus expectedly only tap into shallow, meteoric-sourced fluids near the zone of recharge. The high redox potential and DIC concentrations, as well as low salinity and trace metal content of alkaline peridotite fluids also support the theory of a short residence time and contact with the atmosphere. Surficial aqueous alteration of the host rock would lead to the dissolution of magnesium-bearing phases in peridotite, consequently increasing pH and converting dissolved carbon dioxide to bicarbonate (Neal and Stanger, 1983, 1985; Kelemen et al., 2011). The resulting fluids enriched in magnesium and bicarbonate are consistent with a “Type 1” fluid composition as previously described by Barnes and O'Neil (1969).

Redox potential and DIC concentrations of gabbro-hosted fluids (NSHQ21, WAB103) are similar to those of alkaline-peridotite fluids, implying these fluids also have reacted at a shallow depth while in contact with the atmosphere. However, gabbro-hosted fluids exhibit an order of magnitude higher concentrations of both sodium and chloride, as well as increased concentrations of calcium, silica, aluminum, and trace elements. The increased concentration of calcium and silica in gabbro-hosted compared to alkaline peridotite-hosted aquifers is consistent with previous studies of crustal fluids and likely reflects the mineral composition of gabbros, i.e., more pyroxene and plagioclase feldspar than olivine and serpentine (Stanger, 1986; Dewandel et al., 2005). The high concentrations of sodium and chloride could be derived from leaching of sea salts introduced to the ophiolite during the emplacement of the nappe, mineral dissolution, and/or sea spray (Neal and Stanger, 1985; Stanger, 1986; Murad and Krishnamurthy, 2004; Paukert et al., 2012).

The high conductivity of hyperalkaline peridotite-hosted fluids observed in this study (NSHQ14, WAB71, WAB56) implies extensive water/rock reaction, and the low measured redox potential in combination with the presence of dissolved hydrogen and methane gases is also consistent with the idea that these fluids are the product of low-temperature serpentinization (Neal and Stanger, 1983; Kelemen et al., 2011). The longer residence time predicted for deep groundwater flow would allow for more extended water-rock reaction; continued reaction oversaturates fluids with respect to serpentine and other secondary minerals and further elevates the pH. Magnesium is preferentially sequestered in secondary minerals compared to calcium, resulting in rising calcium concentrations in reacted fluids (Neal and Stanger, 1985). The hyperalkaline pH of these fluids is in accordance with the “Type 2” composition previously described (Barnes and O'Neil, 1969).

There is a marked contrast in the hydrologic properties between the crustal and mantle sequences; hydraulic conductivity is one to two orders of magnitude higher in gabbro-hosted aquifers (Dewandel et al., 2005), potentially leading to the injection of peridotite-hosted fluids into gabbros at the crust-mantle boundary. Local structural features such as faults associated with the crust-mantle boundary may also facilitate mixing of gabbro and hyperalkaline peridotite type fluids (Boudier and Coleman, 1981; Nicolas et al., 2000). This may explain the variable chemical character of some contact fluids (WAB188, WAB55, and NSHQ10). The exception is the well NSHQ4, which despite close proximity to the crust-mantle transition, has a geochemical composition consistent with hyperalkaline peridotite fluids. Analysis of well-chips recovered during drilling of well NSHQ4 also indicate that the subsurface rock-type is entirely peridotite (Miller et al., 2016). Accordingly, we group NSHQ4 with hyperalkaline wells for discussion regarding microbial ecology.

### Influence of aqueous geochemistry on microbial ecology

While there is a clear correlation of fluid type with microbial diversity and structure, it is difficult to statistically discern which geochemical parameters are responsible for controlling the distribution of life in the subsurface of the Samail Ophiolite. This is due to the covariance of geochemical parameters associated with the geologic and hydrologic context of reacted fluids. During water-rock reaction, fluids become progressively more alkaline, reduced, metal-rich, and depleted in dissolved inorganic carbon. For this reason, we only provide speculation for the underlying controls on habitability of this environment.

The dependence of microbial diversity on fluid type is likely due to the availability of particular electron donors and acceptors. Notably, there is a strong imbalance of electron donors and acceptors in most fluids sampled. In hyperalkaline peridotite-hosted fluids in particular, μM to mM concentrations of dissolved hydrogen and methane likely provide significant energy to the subsurface ecosystem. However, while potential electron acceptors such as sulfate and nitrate are present, the concentration of these oxidants is limited and an order of magnitude lower than in alkaline peridotite and gabbro fluids. Conversely, oxidant-rich upper aquifer fluids lack obvious sources of reductants. The presence of dissolved methane in the contact well WAB188 in concert with mM concentrations of sulfate, nitrate, and DIC suggests a favorable niche for microbial life exists at the boundary between peridotite and gabbro where chemical disequilibria derived from the mixing of more oxidized and more reduced fluids likely generates an energy excess. Greater availability of energy sources in contact fluids compared to alkaline peridotite and gabbro-hosted fluids may explain the divergence in community composition of these fluids, despite otherwise fairly similar geochemical compositions.

Serpentinization also yields a unique set of geochemical conditions that organisms inhabiting reacted fluids must adapt to: the most evident consequence being the increase in pH of reacted fluids. The pH of fluids has an apparent negative correlation with microbial diversity as measured by OTU richness (linear regression of pH vs. log_10_ (Richness), multiple *r*^2^ = 0.6604, F-statistic = 35.01, *p* < 0.001; Figure 6) in the range of pH observed in this study. This decrease in richness is most notable at a pH >10. While we do not assume pH to be the sole control on OTU richness, our results suggest that pH may be a good predictor of microbial diversity in a subsurface terrestrial serpentinizing system. These results are consistent with similar observations from the Tablelands and Cedars Ophiolites where lower pH endmember and mixed fluids exhibited greater richness of phylotypes than hyperalkaline endmember fluids (Brazelton et al., 2013; Suzuki et al., 2013). The potential effect of high pH on microorganisms is likely derived from the energy necessary for microorganisms to maintain a proton motive force across the cytoplasmic membrane, a necessity for ATP synthesis (Hicks et al., 2010). Indirectly, pH plays a role in the availability of carbon (Kemmitt et al., 2006; Liu et al., 2014). At a hyperalkaline pH, the primary form of DIC is the carbonate anion, which is considered to be unavailable for carbon fixation for microorganisms (Schrenk et al., 2013; Suzuki et al., 2014). Limited bioavailable inorganic carbon constrains the diversity of organisms that can fix carbon in this setting. Consequently, biological communities in this system may rely on carbon sources other than DIC, such as organic acids, or must possess adaptations to survive.

### The subsurface biosphere inhabiting the samail ophiolite

This study provides the first comprehensive data for subsurface microbial communities in the Samail Ophiolite in Oman, significantly expanding upon the diversity of geochemical regimes investigated compared to the data presented for wells NSHQ14 and NSHQ4 in Miller et al. (2016). We also infer putative metabolisms operating in this subsurface serpentinizing system through comparison of OTUs to closely-related cultivars with known metabolisms, although we have not yet directly assessed the specific activity of the microbial communities detected in this study. Given the dissimilarity of microbial community structure between fluid types, the microbial ecology of each discrete fluid type will be discussed separately below.

#### Ecology of microbial communities inhabiting hyperalkaline fluids

Compounds supplied by water-rock reactions are a potential source of energy for subsurface microbial life in hyperalkaline peridotite fluids. We observed dissolved hydrogen in four wells (NSHQ14, WAB56 and WAB71, NSHQ4), and the maximum measured concentrations were up to 2.88 mM, more than twice the concentration observed in seeps at the Tablelands Ophiolite known to support hydrogen-metabolizing life (Brazelton et al., 2012, 2013; Szponar et al., 2013). The absence of detectable hydrogen in some fluids does not necessarily preclude hydrogen-based metabolism. It is possible that the activity of microorganisms draws down the concentration of this dissolved gas below the limit of detection in this study (0.45 μM), but nM concentrations could potentially be present and sustain life.

Additionally, the presence of hydrogen could support methanogenesis and acetogenesis where DIC concentrations are sufficient. While it is unclear if the methane or acetate detected in these samples is biotic in origin (Miller et al., 2016, 2017; Etiope, 2017), these compounds could in turn support life as additional electron donors for aerobic or anaerobic metabolism. Moreover, organic acids, including formate, detected in μM concentrations in these fluids could be utilized by heterotrophic and fermentative organisms.

Hyperalkaline peridotite fluids contain abundant electron acceptors in the form of sulfate, nitrate, and ferric iron. Sulfate and nitrate were present in μM concentrations (up to 364 and 26 μM respectively) in the fluids, and ample ferric iron is stored within secondary minerals as the byproduct of serpentinization. Such Fe(III) in serpentine and Fe(III) in magnetite in near-surface rocks (Miller et al., 2016) could act as an additional oxidant to sustain life in highly reduced fluids (Ménez et al., 2012). These observations are in contrast to the current perception that terrestrial serpentinizing systems are inherently limited in electron acceptors (Schrenk et al., 2013).

The microbial community structure of deep, hyperalkaline fluids of the Samail Ophiolite differs from the communities studied at surficial hyperalkaline seeps in ophiolitic sites worldwide thus far. In Oman, hyperalkaline peridotite-hosted fluids are dominated primarily by members of the family Thermodesulfovibrionaceae, candidate phylum OP1 class MSBL6, and *Meiothermus*.

The sulfate-reducing family Thermodesulfovibrionaceae is a deeply-branching bacterial clade in the phylum Nitrospirae (Henry et al., 1994). Members of this family can be chemoorganotrophic or chemolithoautotrophic, reducing sulfate coupled to the oxidation of hydrogen or C1–C3 acids (Henry et al., 1994; Sekiguchi et al., 2008). Some members alternatively may use ferric iron or nitrate as terminal electron acceptors for anaerobic respiration (Sekiguchi et al., 2008). This family was detected in the Zambales Ophiolite of the Philippines. However, less than 0.2% of sequence reads were affiliated with Thermodesulfovibrionaceae (Cardace et al., 2015; Woycheese et al., 2015).

Acetogenesis using the reductive acetyl-CoA (or Wood-Ljungdahl) pathway has been proposed as a putative metabolism for members of candidate phylum OP1, or *Candidatus* Acetothermia, although only a few partial genomes from this phylum have been assembled (Takami et al., 2012; Badhai et al., 2015; Hu et al., 2016). This pathway has been found to be prevalent in other oligotrophic, deep crystalline aquifers such as the Witwatersrand basin in South Africa (Müller, 2003; Magnabosco et al., 2016). However, acetogens must compete with other hydrogenotrophs in this environment such as methanogens. The reductive acetyl-CoA pathway is used by both methanogenic archaea and acetogenic bacteria for carbon fixation, but acetogens have a higher threshold for hydrogen than most methanogens (le Van et al., 1998; Berg et al., 2010). This is compounded by the lower energy yield for the conversion of carbon dioxide and hydrogen to acetate than to methane (Thauer et al., 1977; Seifritz et al., 1993; Schink, 1997; Ragsdale and Pierce, 2008). Accordingly, many acetogens possess versatile metabolic capabilities including the usage of a variety of alternative electron acceptors such as nitrate (Seifritz et al., 1993; Ragsdale and Pierce, 2008; Lever, 2012). OP1 is not prevalent in any other terrestrial serpentinizing system, but was detected in high abundance in carbonate chimneys from the meteorically-sourced, serpentinization-driven, submarine hydrothermal system of Prony Bay, New Caledonia (Monnin et al., 2014; Postec et al., 2015).

*Meiothermus* is an aerobic heterotrophic genus belonging to the Deinococcus/Thermus clade (Albuquerque et al., 2009; Tindall et al., 2010). Taxa from this group have been detected at the Lost City Hydrothermal Field and the Cabeço de Vide aquifer (CVA) in Portugal, but the genus *Meiothermus* has only been detected at two other serpentinizing sites: at Manleluag in the Zambales Ophiolite of the Philippines and at the hydrothermal field at Prony Bay (Brazelton et al., 2010; Tiago and Veríssimo, 2013; Woycheese et al., 2015; Mei et al., 2016). Interestingly, this aerobic genus was only present at greater than 0.5% relative abundance in hyperalkaline peridotite-type fluids, which are characterized by highly negative measured redox potentials. This is in contrast to the Zambales Ophiolite where the greatest relative abundance of *Meiothermus* was observed in the most well-mixed, oxygenated fluid end-member (Woycheese et al., 2015).

The predominance of the aerobic heterotroph *Meiothermus* in conjunction with obligate anaerobes such as Thermodesulfovibrionaceae is rather peculiar. The co-occurrence of strictly aerobic and anaerobic microorganisms in sampled fluids suggests a mixing of deep and shallow well fluids during pumping for sample acquisition. Near-surface fluids in the well likely have had more recent interaction with atmospheric oxygen. We did flush stagnant well-water through pumping out a well volume prior to sampling. However, intervals of variable permeability may exist in the well that tap into fluids of varying residence time, resulting in variation in redox potential with depth down-well. If an upper zone of oxidized fluids does exist, the interface between these oxidized and reduced fluids could provide chemical disequilibria that could stimulate the growth of organisms such as *Meiothermus*. Future sampling using packer systems to isolate fluids from specific depth intervals will provide insight into microbial diversity down-well.

Alternatively, detected aerobic organisms such as *Meiothermus* may be genuine members of the deep subsurface biosphere, but respire very slowly or function as a facultative organism that can use alternate electron acceptors such as nitrate. Oxygen could be introduced into subsurface fluids through atmospheric sources via fracture networks or produced in small quantities through subsurface reactions such as the radiolysis of water (Lin et al., 2005a; Purkamo et al., 2016). Gas-tight sampling of deep fluids for accurate oxygen measurements would be necessary to predict the thermodynamic feasibility of aerobic metabolism in these deep fluids. It is also possible that the OTU affiliated with *Meiothermus* detected in this system represents a novel strain capable of anaerobic respiration. Future efforts to culture or assemble the genomes of the strains of *Meiothermus* present in these wells could help to confirm or reject this hypothesis.

The predominance of Thermodesulfovibrionaceae, candidate phylum OP1, and *Meiothermus* indicates that the subsurface biosphere of the Samail Ophiolite is distinct compared to previously characterized terrestrial ophiolites. In contrast, the abundance of organisms such as those affiliated with Anaerobrancaceae and the order Clostridiales in hyperalkaline fluids suggests a close similarity with other oligotrophic, hydrogen-enriched, rock-hosted ecosystems. Purkamo et al. (2016) discovered phylotypes affiliated with Anaerobrancaceae, Pseudomonadaceae, Comamonadaceae, and Firmicutes were common to all deep crystalline bedrock fracture fluids at Outokumpu. We also detect OTUs belonging to these clades in all hyperalkaline wells, although the basis for the ubiquity of these organisms as well as their potential functional roles in the subsurface remains unclear.

The family Anaerobranceae is obligately anaerobic and includes multiple alkaliphilic strains, including species that are capable of metal and sulfur reduction (Prowe and Antranikian, 2001; Gorlenko et al., 2004; Kevbrin et al., 2008). This family was also found to be prevalent in the serpentinite-hosted hydrothermal field at Prony Bay (Mei et al., 2016). The potential function of Comamonadaceae in the deep subsurface is much more ambiguous; members of this family have diverse metabolisms including denitrification, ferric iron reduction, hydrogen oxidation, and fermentation (Willems and Gillis, 2015). We detected one OTU affiliated with this family that was present in all fluid samples. We additionally detected an OTU assigned to the genus *Hydrogenophaga*, a hydrogen-oxidizer common in serpentinizing ecosystems (Brazelton et al., 2012, 2013; Suzuki et al., 2013; Woycheese et al., 2015).

From the phylum Firmicutes, we detected multiple OTUs including phylotypes assigned to the order Clostridiales and the genus *Desulfotomaculum*. Members of the genus *Desulfutomaculum* were previously detected at the serpentinizing hydrothermal fields of Lost City and Prony Bay and are typically sulfate-reducers that oxidize hydrogen or organic compounds, although some strains instead produce hydrogen during fermentation through a syntrophic relationship with methanogens (Brazelton et al., 2006, 2010; Imachi et al., 2006; Mei et al., 2016). The order Clostridiales has also been detected at numerous serpentinizing sites and deep crystalline aquifers (Brazelton et al., 2006, 2012; Lin et al., 2006; Suzuki et al., 2013; Tiago and Veríssimo, 2013; Quéméneur et al., 2014; Postec et al., 2015; Woycheese et al., 2015; Mei et al., 2016). The universal presence of the phylum Firmicutes in serpentinizing settings implies these organisms are well-adapted to the extreme conditions of reacted fluids including a hyperalkaline pH, a limited concentration of dissolved inorganic carbon, and a highly reduced redox state. Although, the abundance of sequences from a spore-forming phyla raises the question of whether or not Firmicutes detected under these extreme conditions are actively metabolizing cells or are instead inactive endospores. The formation of endospores could allow survival of these organisms in an environment with only sporadic fluxes of energy. Accordingly, the activity of this clade will need to be investigated further using a comprehensive approach, e.g., fluorescent *in situ* hybridization of vegetative cells, transcriptomics, and detection of endospores through staining and quantification of dipicolonic acid (Kieft, 2016).

In addition to these common clades, we also detected the presence of *Methanobacterium*, a methanogen that generates methane using hydrogen with formate, CO, or carbon dioxide (Balch et al., 1979). The genus *Methanobacterium* was first detected in Oman in 2014 in well NSHQ4, which contained millimolar concentrations of methane with an enigmatic, positive δ^13^C signature (Miller et al., 2016). *Methanobacterium* has also been observed in the Zambales and Del Puerto ophiolites, as well as in other deep, rock-hosted systems (Moser et al., 2005; Blank et al., 2009; Woycheese et al., 2015; Purkamo et al., 2016). Methanogens have been proposed as keystone species for hydrogen-driven ecosystems because even at a low abundance, these organisms may play a large role in primary productivity for the entire ecosystem (Pedersen, 2000; Nealson et al., 2005; Purkamo et al., 2016). We do detect the co-occurrence of the aerobic methanotroph *Methylococcus capsulatus* in all hyperalkaline fluids in Oman (Miller et al., 2016; this study), suggesting methane produced by methanogens may support other microorganisms *in situ*. Remarkably, we do not detect the presence of anaerobic methanotrophs belonging to the ANME group as observed at other serpentinizing sites, despite the reduced nature of sampled fluids and co-existence of methane and sulfate (Brazelton et al., 2006; Suzuki et al., 2013; Tiago and Veríssimo, 2013; Quéméneur et al., 2014; Postec et al., 2015). It is possible that anaerobic methanotrophy could be coupled to other electron acceptors such as nitrite. We did detect the denitrifying methanotrophic bacterial genus *Candidatus* Methylomirabilis in 2.88% relative abundance in the alkaline peridotite well WAB104 (Ettwig et al., 2010; Wu et al., 2011). However, this genus was absent in hyperalkaline fluids. It remains unclear whether other organisms not yet known to oxidize methane anaerobically are occupying this niche.

Altogether, we observe an interesting mixture of chemoorganotrophic and chemolithotrophic organisms that use a wide range of substrates for biosynthesis. These communities likely utilize endogenous energy sources such as hydrogen for acetogenesis and methanogenesis, but fermentation and heterotrophy using sulfate, nitrate, or ferric iron also appear to be common in these deep, hyperalkaline fluids.

#### Microbial ecology of upper aquifer fluids

Potential electron donors in alkaline peridotite and gabbro fluids with no detected hydrogen or methane are more enigmatic. Mineral sources may provide a source of energy in the form of ferrous iron or ammonium (Hirayama et al., 2005; Cardace et al., 2015). Reduced iron remains in partially-serpentinized harzburgites and dunites from the Samail Ophiolite, and thus could be available for microbial oxidation (Miller et al., 2016). Ammonium is common in deep subsurface fluids, although the source is typically unknown. Mineral hosts for ammonium are not often rigorously identified. However, ${\text{NH}}_{4}^{+}$ can be incorporated into mantle clinopyroxene (Watenphul et al., 2010) or substitute for K^+^ in phyllosilicates, giving rise to ${\text{NH}}_{4}^{+}$ release during water rock interaction (Holloway and Dahlgren, 2002; Swanner and Templeton, 2011). Alternatively, ${\text{NH}}_{4}^{+}$ can be derived through *in situ* biological fixation. It is also possible that organic electron donors may be essential for metabolism in these fluids. While we did not measure total dissolved organic carbon in fluids, all wells contained μM concentrations of formate and acetate, and some fluid samples also contained lactate, butyrate, propionate, or valerate. Although the source is not well constrained, this dissolved organic carbon could support the growth of both heterotrophic and fermentative microorganisms. Nitrate and sulfate are abundant electron acceptors, and are present at an order of magnitude greater concentration (up to 3893 and 356 μM respectively) than in hyperalkaline fluids. While the sources of these electron acceptors are enigmatic, the depletion of these e-acceptors in deeper fluids can likely be attributed to their consumption by nitrogen and sulfur-cycling microorganisms in the upper aquifer.

Microbial nitrogen-cycling, particularly nitrification, appears be a significant biogeochemical process in upper aquifer fluids. The family Nitrososphaeraceae of the archaeal phylum Thaumarchaeota are involved in the aerobic oxidation of ammonia to nitrite for autotrophic growth (Stieglmeier et al., 2014). The order Nitrospirales is also prevalent in these fluids; we detected both the genus *Nitrospira* and the family 0319-6A-21 in all upper aquifer fluids sampled. The genus *Nitrospira* participates in the second step of nitrification by oxidizing nitrite to nitrate, although some members of this genus have been found to possess the genes for both steps of nitrification, completely oxidizing ammonia to nitrate (Daims et al., 2015). *Nitrospira* strains are highly flexible, and have been found to have the capability to utilize various organic compounds in lieu of chemolithotrophic growth with nitrite, and even oxidize hydrogen aerobically when enough oxygen is present (Koch et al., 2014, 2015). Thus, it is not clear if these organisms are actively involved in subsurface nitrification. Nitrifiers have been detected at other sites where serpentinization is thought to be taking place; the presence of Thaumarcheota was reported at Prony Bay, and Nitrospirae at seeps at CVA and the Cedars, although not in fluids correlated with the source (Suzuki et al., 2013; Tiago and Veríssimo, 2013; Postec et al., 2015). With the exception of the contact well WAB55, nitrifiers were not abundant in any other fluid type. We did not measure ammonium concentrations in these fluids; however, both nitrate and nitrite were above detection limit in all upper aquifer fluids, supporting the assertion that upper aquifer fluids experience microbial nitrification.

Gabbro and alkaline peridotite-hosted fluids are also enriched in candidate phyla Parvarcheota and OP3. The order YLA114 was present in >1% relative abundance in all upper aquifer samples. The order YLA114 was first detected in the hydrogen-enriched, circumneutral Yellowstone Lake (Kan et al., 2011). This candidate division consists of ultrasmall, uncultivated microorganisms that are predicted to have an aerobic metabolism (Baker et al., 2010). This division has been detected in diverse environments including geothermal calcite microbialites in Ciocia, Romania, volcanic lakes in Poás, Costa Rica, and metal-rich tropical stream sediment in Brazil suggesting that these organisms are adapted to extreme environments (Cabassi et al., 2014; Coman et al., 2015; Costa et al., 2015). Additionally, we detected the candidate division OP3, recently named “Omnitrophica” belonging to the Planctomycetes/Verrucomicrobia/Chlamydia (PVC) superphylum; this candidate phylum has been frequently reported in anaerobic settings associated with redox cycling of iron, heavy metals, and sulfur (Glöckner et al., 2010; Fuerst, 2013; Rinke et al., 2013). The potential function of these organisms in the environment, however, remains enigmatic.

As we reported for hyperalkaline peridotite-hosted fluids, there was also a perplexing mix of inferred aerobic and anaerobic organisms detected in upper aquifer fluids. This observation is likely due in part to the use of a submersible pump, as discussed previously. However, a co-occurrence of genes encoding for ammonia oxidation and denitrification have been described in oceanic crustal gabbros and basalts (Mason et al., 2008, 2010). This coexistence was attributed to potential “microniches” within rocks on a small spatial scale (Mason et al., 2008). Thus, it is possible that ammonia-oxidizing Nitrosospharaceae and acetogenic *Cand*. Acetothermales could actively metabolize within discrete zones in stored fluids and rock matrices tapped by the same well.

#### Contact fluids: unique niche for microbial life in terrestrial ophiolites

Contact fluids do not host a consistent core microbial community structure that is common to all contact wells; however, the prevalence of betaproteobacterial OTUs is one universal component of contact fluid ecosystems. The phylotypes affiliated with Betaproteobacteria do vary considerably well to well. WAB188 contains up to a 20.76% relative abundance of the genus *Sulfuritalea*, consisting of autotrophic microorganisms that can oxidize thiosulfate, elemental S, or hydrogen as sole energy sources for growth (Kojima and Fukui, 2011). This phylotype was also observed in NSHQ10, but only at 4.38% relative abundance, and has also been observed at the hydrothermal field of Prony Bay (Postec et al., 2015). NSHQ10 is dominated (33.71% relative abundance) by a Betaproteobacterial phylotype affiliated with the Rhodocyclaceae genus Dok59. The family Rhodocyclaceae is associated with diverse metabolic functions; members of this family can degrade a wide range of carbon sources using various electron acceptors including nitrate, chlorate, perchlorate, and selenate (Oren, 2014). This family also contains chemoautotrophic sulfur oxidizers, methylotrophs, and nitrogen fixers, and thus the function of this family in this environment is unknown (Oren, 2014). In WAB55 in the year 2015, 45.61% of the community consisted of the genus *Cupriavidus*. This genus also has flexible metabolic capabilities including the autotrophic oxidation of hydrogen, iron, and sulfur and heterotrophy (aerobically and anaerobically through denitrification) and some strains are highly resistant to heavy metals (Vandamme and Coenye, 2004; Janssen et al., 2010; Shelobolina et al., 2012).

We also observe other organisms likely involved in the biogeochemical cycling of sulfur, nitrogen, and iron in contact wells. The sulfate-reducing Clostridial family Desulforudaceae was present in all contact fluids, and constituted 12.18% relative abundance in WAB188 in the 2015 field season. The order Desulforudales, to which the family Desulforudaceae belongs, was also detected in deep bedrock fracture fluids from the Fennoscandian Shield associated with the sulfate-methane interphase (Bomberg et al., 2015). Interestingly, the high relative abundance of Desulforudaceae in WAB188 in 2015 was also accompanied by 8.89% relative abundance of the methanogen *Methanobacterium* implying a similar relationship. A phylotype affiliated with Thermodesulfovibrionaceae genus LCP6 was also detected in NSHQ10; this phylotype is different than the Thermodesulfovibrionaceae phylotype observed in hyperalkaline peridotite fluids, although that same phylotype was observed in lesser abundance in all contact fluids. Together, the presence of organisms such as Desulforudaceae and Thermodesulfovibrionaceae imply sulfate reduction is an important metabolic process in contact fluids. We additionally observed evidence for nitrogen cycling in contact fluids, particularly in the well WAB55. In WAB55, the order Brocadiales family W4 was present in 5.33% relative abundance in the year 2016. The order Brocadiales consists of anaerobic ammonium-oxidizing (anammox) bacteria that derive energy for growth through the conversion of ammonium and nitrite into nitrogen gas under anoxic conditions (Jetten et al., 2009). This same well also had the nitrifying genus *Nitrospira* present in 15.28% relative abundance, indicating that nitrogen cycling is particularly active under at least some contact fluid conditions. Potential nitrite or iron-reducers belonging to the phylum Chlorobi are also prevalent in the well WAB188. These organisms belong to the family Melioribacteraceae, members of which are chemoorganotrophs that can respire anaerobically through reduction or ferric iron, nitrite, and arsenate, but can also utilize fermentation or aerobic respiration for metabolism (Podosokorskaya et al., 2013). We also detect the Chlorobi BSV26 clade in high abundance (7.41% relative abundance in 2016) in WAB55, although the metabolism of this clade is not yet known.

The prevalence of Betaproteobacteria in contact fluids bears resemblance to other rock-hosted systems, including ophiolites. In the Fennoscandian Shield, the Witwatersrand Basin, and in serpentinizing seeps of terrestrial ophiolites, there is an evident pattern in microbial community structure where Proteobacteria are more dominant in shallower, more oxygenated groundwater, and Firmicutes dominate in the deeper, anoxic fluids (Itävaara et al., 2011; Brazelton et al., 2013; Suzuki et al., 2013; Magnabosco et al., 2016). In the Samail Ophiolite, contact wells likely represent zones in the aquifer where more oxidizing, shallow fluids mix with deeper, anoxic fluids. Betaproteobacteria, in particular, seem to thrive under these mixing conditions. While we did not observe the Betaproteobacterial genus *Hydrogenophaga* prevalent in the Cedars and Tablelands ophiolites, there was an abundance of other genera in contact fluids that require reductants such as hydrogen from deep fluids and oxidants from surface inputs. The chemical disequilibria between these two fluid types should provide abundant energy for life, as evidenced by the greater richness observed in contact fluids compared to deeper sourced, hyperalkaline fluids. Accordingly, contact zones offer a unique niche in the aquifer for microbial life.

#### Ubiquitous phylotypes

Unclassified bacteria, candidate phyla OD1, GAL15, and OP1, as well as Betaproteobacteria belonging to the family Comamonadaceae were ubiquitous across fluid types. The candidate division OD1 has primarily been detected in anoxic environments, and a partial genome from this division includes genes that have been identified in anaerobic organisms, suggesting members of this division are likely anaerobic (Elshahed et al., 2005; Briée et al., 2007; Barberán and Casamayor, 2011; Peura et al., 2012). Similarly, little is known regarding members of GAL15; however, these organisms have been detected in other extreme environments such as nutrient poor, hyperarid fumarole deposits and metal and radionuclide contaminated subsurface sediment samples (Costello et al., 2009; Lin et al., 2012). The candidate phylum OP1, or *Candidatus* “Acetothermia” as proposed by Rinke et al. (2013), is predicted to be one of earliest evolved bacterial chemolithoautotrophic lineages (Takami et al., 2012). A nearly complete genome of *Candidatus* “Acetothermus autotrophicum” has been assembled containing genes encoding for a likely acetogenic metabolism (Takami et al., 2012). The universal detection of *Cand*. Acetothermia in all sampled wells suggests acetogenesis could be a widespread metabolism in the serpentinizing subsurface; although, it is possible that other members of this candidate division may possess genes for alternative metabolic capabilities. And while the family Comamondaceae is well-characterized, the OTU detected in all fluid samples (Supplemental Table 2) was not classified at the genus level. This family harbors remarkable metabolic diversity with genera involved in denitrification, ferric iron reduction, hydrogen oxidation, and fermentation, and thus the role this phylotype may play in serpentinizing environments is not evident (Willems and Gillis, 2015). Future microbiological studies of subsurface fluids from Oman may provide further insight into the functional role of these microorganisms, especially if these organisms can be cultured for laboratory study.

## Conclusions

In the actively serpentinizing Samail ophiolite, we identify four habitable subsurface environments hosting unique microbial communities. The peridotite and gabbro aquifers store fluids with highly variable amounts of dissolved hydrogen, methane, and inorganic carbon, as well as pervasive low micromolar concentrations of formate and acetate as alternative reductants and carbon sources. Oxidants are abundant and include sulfate, nitrate, nitrite, and Fe(III) minerals. The appreciable concentrations of electron acceptors in all fluids suggests that the serpentinized subsurface may not be as limited in oxidants as previously thought. For example, our data suggest that sulfate is a critical electron acceptor for sustaining subsurface microbial life within the ophiolite. Active nitrogen cycling also occurs, and processes such as ammonium oxidation, nitrite oxidation, and nitrate reduction may be strongly controlled by the hydrologic regime.

Comparisons of the microbial community structure and diversity of deep gabbro- and peridotite-hosted fluids in Oman offers a framework for differentiating distinct subsurface microbial biomes. We found that the diversity of detected microbial communities varies considerably with fluid type. pH is a predictor for the richness of microbial communities. Microbial communities in reacted, hyperalkaline peridotite fluids are characterized by low richness; these communities differ from those previously detected at hyperalkaline springs from ophiolite systems worldwide and are comprised primarily of *Meiothermus*, Thermodesulfovibrionaceae, and candidate phylum OP1 class MSBL6. However, we also detect abundant Comamonadaceae, including *Hydrogenophaga*, as well as Firmicutes, Psuedomonadaceae, and *Methanobacterium*, indicating a core similarity to other deep subsurface or serpentinizing settings. From these taxonomic affiliations, we infer methanogenesis, acetogenesis, and fermentation are dominant metabolisms in highly reacted fluids. Hydrogen metabolism is likely also coupled to several strong oxidants such as sulfate, Fe(III)-minerals, and nitrate.

Gabbro and alkaline peridotite fluids host relatively rich microbial communities dominated by candidate phyla as well as phylotypes affiliated with nitrification, including Nitrosospharaceae and *Nitrospira*, suggesting nitrogen cycling may be particularly active in upper aquifer fluids. We observe a unique community inhabiting wells at the contact between gabbro and peridotite; these communities do not host a consistent, core microbial community, but are particularly enriched in Betaproteobacteria. We hypothesize these contact zones represent areas of fluid mixing, enhancing chemical disequilibrium. The consistent parsing of microbial communities as a function of fluid type is likely driven by differential energy availability and aqueous geochemistry (e.g., pH, Eh, available DIC and electron acceptors) that results from fundamental differences in both host rock lithology and the extent of water-rock reaction. Thus, identifying the origin and reaction paths of fluids, as well as the source and rates of consumption of oxidants and reductants in such systems, is integral to understanding the limits to life in the subsurface of terrestrial ophiolites.

This work provides an important framework for interpreting the habitability of terrestrial serpentinizing environments, and raises many additional questions. These communities should be targeted for physiological and field-based studies to identify the energetic costs and mechanisms of organisms capable of surviving under extreme energy limitation in rock-hosted environments. Further research is necessary to quantify the biomass residing in this ecosystem and to determine how the rates of key metabolic processes are distributed across geochemical gradients. Thermodynamic modeling of energy densities in this system could provide testable insights into the potential activity and metabolisms of this ecosystem, particularly if combined with genomic data. This work would be especially beneficial in determining the efficiency and prevalence of heterotrophic vs. chemoautotrophic metabolisms that may operate in this environment. Notably, both organic and inorganic electron donors are available, even in deep hyperalkaline fluids. Elucidating how the coupled hydrology and water/rock reactions generate the flux of electron donors and acceptors in this system is also essential to developing an understanding of the subsurface microbial ecology of terrestrial serpentinites. Isotopic measurements of these chemical constituents and their possible sources could resolve the question of their origin, further constraining how the distribution of life in this subsurface ecosystem varies across geologic context and hydrologic regime. All of these efforts will be greatly aided by increased access to fluids and their host rocks for repeated biogeochemical and microbiological sampling, monitoring, and experimentation through the International Continental Drilling Program (ICDP) project in Oman (http://www.omandrilling.ac.uk/), which will provide a tractable experimental system for forthcoming subsurface biosphere research.

## Author contributions

AT, JM, KR, HM, and PK conceived the study, organized access to the boreholes and conducted initial site assessments and sample collection. KR, HM, DN, NB, JM, NF, and AT collected and/or analyzed samples and/or assisted in the data interpretation. KR wrote the manuscript; all authors critically revised the manuscript text and figures.

## Funding

The biological research was funded by the Rock-Powered Life NASA Astrobiology Institute (Cooperative Agreement NNA15BB02A), with supporting geochemical work funded through the Department of Energy (DE-SC0006886). We would like to thank the Alfred P. Sloan Foundation Grant 2014-3-01 to PK for additional support for field logistics associated with this work.

### Conflict of interest statement

The authors declare that the research was conducted in the absence of any commercial or financial relationships that could be construed as a potential conflict of interest.
