# Supplementary material for: Geological and Geochemical Controls on Subsurface Microbial Life in the Samail Ophiolite, Oman
Source: Front Microbiol. 2017 Feb 7;8:56. doi: 10.3389/fmicb.2017.00056 (PMC5293757; doi:10.3389/fmicb.2017.00056)
Supplement: Supplementary file 3 [file DataSheet1.docx]

Supplementary Material

Geological and Geochemical Controls on Subsurface Microbial Life in the Samail Ophiolite, Oman

Kaitlin R. Rempfert^*^, Hannah M. Miller, Nicolas Bompard, Daniel Nothaft, Juerg M. Matter, Peter Kelemen, Noah Fierer, Alexis S. Templeton^*^

*** Correspondence:** Kaitlin Rempfert: [kaitlin.rempfert@colorado.edu](mailto:kaitlin.rempfert@colorado.edu); Alexis Templeton: [alexis.templeton@colorado.edu](mailto:alexis.templeton@colorado.edu)

#

# Supplementary Figures

**
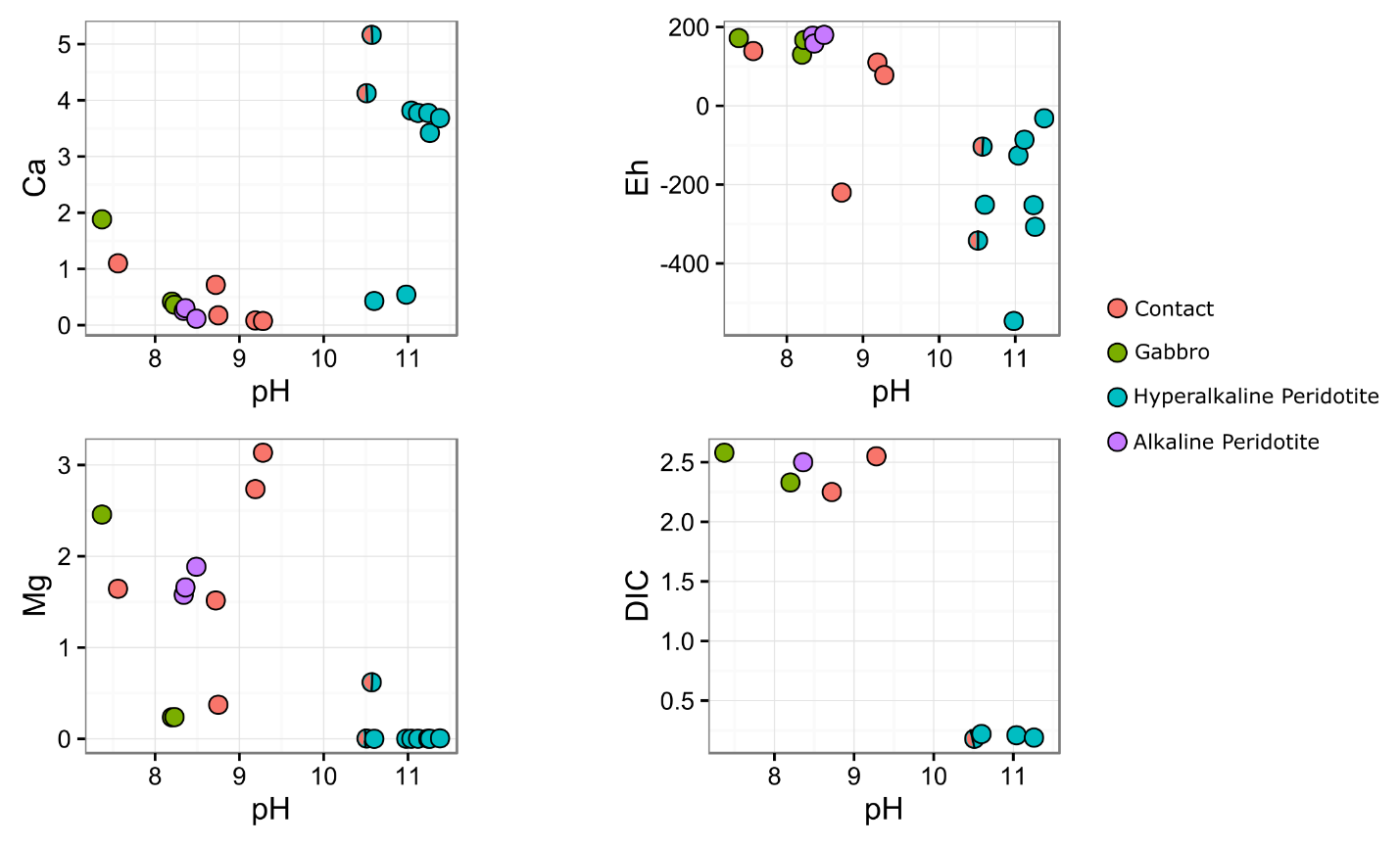
**

**Supplemental Figure 1:** Crossplots of calcium (A), Eh (B), magnesium (C), and DIC (D) versus pH. Concentrations of calcium, magnesium, and total dissolved inorganic carbon (measured as dissolved carbon dioxide) are reported in mM. Eh is reported in mV. Wells are color-coded based on fluid type, with the well NSHQ4 depicted as both a contact well and a hyperalkaline peridotite well (based on geologic context, this well is classified as contact, however, NSHQ4 has fluid chemistry consistent with hyperalkaline peridotite wells). Generally, with increasing pH, DIC and magnesium concentrations decrease, calcium concentrations increase, and more negative Eh values are observed.
